# Nanostructured surfaces for enveloped virus inactivation: mechanisms and fabrication strategies

**DOI:** 10.3389/fbioe.2026.1885559

**Published:** 2026-07-01

**Authors:** Jiayu Wang, Nana Zhou, Jiarui Qu, Rui Sun

**Affiliations:** 1 Pediatric Outpatient Department, The First Hospital of Jilin University, Changchun, China; 2 Critical Care Medicine Nursing Platform, The First Hospital of Jilin University, Changchun, China; 3 Research Department, The First Hospital of Jilin University, Changchun, China

**Keywords:** antiviral, mechanical piercing, nanostructured surfaces, stretching-induced rupture, surface engineering

## Abstract

Enveloped viruses, owing to the intrinsic sensitivity of their lipid envelopes to physical perturbations, have attracted increasing attention in the field of antiviral surface engineering. Compared with conventional chemical-based strategies, nanostructured surfaces can inactivate viruses through interfacial physical interactions without the need for continuous external stimuli. This review summarizes the primary mechanisms underlying nanostructure-induced viral inactivation, including mechanical piercing, stretching-induced rupture, and array-induced synergistic effects. Among these, membrane stretching and localized stress concentration arising from multivalent contact are identified as the dominant mechanisms. Key factors influencing antiviral performance, such as structural spacing, tip geometry, and virus size, are further discussed. In addition, common fabrication approaches, including etching, template-assisted replication, and plasma-based techniques, are reviewed. The contributions of surface composition to antiviral performance are also highlighted, with emphasis on the synergistic effects between nanostructured features and functional materials. Finally, current challenges related to mechanistic understanding, structure–activity relationships, and practical implementation are highlighted, providing insights for the rational design of antiviral surfaces.

## 1 Introduction

Enveloped viruses are characterized by an outer lipid bilayer membrane and include important human pathogens such as influenza viruses and coronaviruses (Bar-On et al., 2020; Fehr and Perlman, 2015). This lipid envelope plays a critical role in viral infection processes, including host cell recognition and membrane fusion, and its structural integrity directly determines viral infectivity (White and Whittaker, 2016; Sanders et al., 2021). At the same time, the presence of the lipid envelope renders these viruses more susceptible to physical and chemical perturbations compared to non-enveloped viruses, making them attractive targets for antiviral surface engineering (Aboubakr et al., 2021; Kampf et al., 2020a).

Conventional strategies for inactivating enveloped viruses primarily rely on chemical disinfectants, metal ion release, or photocatalytic reactions, which disrupt the lipid membrane or oxidize viral components (Kampf, 2020b; Alavi et al., 2022; Zhang et al., 2019; Birkett et al., 2022). However, these approaches often suffer from limited durability, strong dependence on environmental conditions, and potential toxicity, thereby restricting their application in long-term protection scenarios. Consequently, there is growing interest in developing alternative strategies that enable virus inactivation through interfacial physical interactions without the need for continuous external stimuli.

In recent years, nanostructured surfaces have demonstrated unique potential for modulating bio-nano interactions owing to their tunable geometries and interfacial properties (Linklater et al., 2021; Hasan et al., 2013; Anselme et al., 2010; Birkett et al., 2022). By designing nanostructures with specific sizes, spacings, and curvatures, it is possible to introduce localized stress concentrations and geometric constraints during virus-surface contact, thereby affecting viral structural stability (Linklater et al., 2023). Previous studies have shown that when the characteristic dimensions of nanostructures are comparable to viral size, enveloped viruses can experience significant deformation and tension imbalance under multivalent contact conditions, ultimately leading to disruption of the lipid envelope and reduced infectivity (Nagarajan et al., 2021). This morphology-induced physical mechanism provides a promising route for constructing antiviral surfaces without relying on chemical release.

Despite these advances, the underlying mechanisms governing nanostructure-virus interactions remain insufficiently understood (Jaggessar et al., 2022). Current studies are largely limited to phenomenological observations and qualitative interpretations, and a unified understanding of the quantitative relationships between structural parameters and antiviral performance is still lacking. Furthermore, variations in size, structure, and mechanical properties among different enveloped viruses may lead to distinct response behaviors. Therefore, a systematic review of nanostructure-mediated viral inactivation is needed to clarify the underlying mechanisms, establish design principles, evaluate fabrication strategies and engineering challenges, and highlight the contributions of surface composition and its synergistic interactions with nanostructures. Such an overview will provide valuable guidance for the development of efficient antiviral functional surfaces.

## 2 Mechanisms and key influencing factors of nanostructure-induced antiviral effects

The antiviral performance of nanostructured surfaces primarily arises from their ability to mechanically disrupt the viral envelope. The underlying mechanisms can be broadly categorized into mechanical piercing, stretching-induced rupture, drying-induced damage, and array-induced synergistic effects.

**FIGURE 1:**
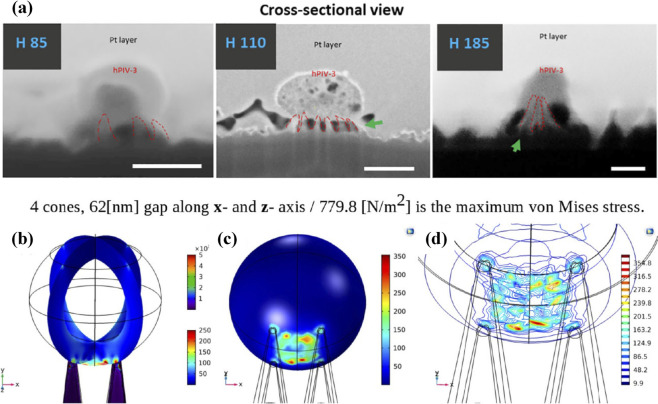
Mechanisms of nanostructure-induced antiviral effects. **(a)** Focused ion beam–scanning electron microscopy images showing deformation of hPIV-3 on nanopillars with varying heights (scale bar: 200 nm). Reproduced with permission from ref (Mah et al., 2026). Copyright 2026 Wiley-VCH GmbH. **(b)** Finite element simulation of a spherical virus interacting with four nanocones (spacing: 62 nm), illustrating stress buildup at the virus bottom. **(c)** 3D visualization of von Mises stress distribution. **(d)** Heat map of stress concentration between nanocone tips. Reproduced with permission from ref (Mah et al., 2024). Copyright 2024 American Chemistry Society.

The mechanical piercing mechanism proposes that highly sharp nanostructures can directly penetrate the viral envelope and compromise its structural integrity (Mah et al., 2026; Mah et al., 2024). Electron microscopy observations have indeed revealed the insertion of nanostructure tips into viral particles, accompanied by local deformation (Figure 1a) (Mah et al., 2026). However, theoretical studies suggest that penetration of a lipid bilayer requires a substantial energy input (on the order of ∼100 kT), indicating that contact forces alone are generally insufficient to induce spontaneous piercing. Therefore, this mechanism is more likely to occur under the assistance of external forces or pre-existing membrane tension, rather than serving as the dominant pathway.

In contrast, stretching-induced rupture is recognized as the primary mechanism governing nanostructure-mediated antiviral activity (Mah et al., 2026; Mah et al., 2024). When viruses adhere to nanostructured arrays, multivalent contacts are established between the viral envelope and multiple nanostructures, effectively suspending the virus across adjacent features. Under interfacial adhesion forces, the viral membrane experiences global stretching, while pronounced local stress concentrations develop at the nanostructure tips (Figures 1b–d); (Mah et al., 2024). Once the membrane tension exceeds its mechanical threshold, pore formation or catastrophic rupture occurs. Finite element simulations have demonstrated that antiviral efficacy relies on the collective action of multiple nanostructures rather than on localized penetration by a single feature (Mah et al., 2026).

In addition, array-induced synergistic effects significantly enhance antiviral performance (Wang et al., 2023). Compared with isolated nanostructures, ordered arrays provide increased contact points, leading to greater overall membrane deformation and more distributed stress fields. Furthermore, nanostructures with smaller feature sizes, particularly reduced tip diameters, generate stronger local stress concentrations, thereby accelerating membrane deformation and failure. The collective interaction within the array not only increases the effective penetration depth but also amplifies the rate and extent of membrane disruption.

From a design perspective, the geometric parameters of nanostructures play a dominant role in determining antiviral performance. Among these, the interstructure spacing (pitch) is the most critical factor: smaller spacing promotes multivalent contact and enhanced membrane stretching, whereas larger spacing typically results in single-point interactions and markedly reduced antiviral efficacy (Mah et al., 2026). In contrast, the effect of structure height is conditional; increased height can enhance deformation at intermediate spacing, but becomes less significant at larger pitches (Mah et al., 2026). Tip geometry also plays a decisive role, as smaller tip radii induce higher local stress concentrations and improve antiviral efficiency (Mah et al., 2024; Wang et al., 2023). In addition, virus size influences the interaction outcome, with larger viruses more likely to engage multiple nanostructures simultaneously, thereby facilitating stretching-induced rupture (Mah et al., 2026). Finally, external environmental factors, such as drying processes, can introduce additional tension and further enhance viral inactivation (Shigetoh et al., 2025).

In summary, nanostructure-mediated antiviral activity can be understood as a mechanically driven process governed by multivalent stretching, localized stress concentration, and environmental modulation. Among these factors, interstructure spacing and array-induced synergy play decisive roles, providing key guidelines for the rational design of antiviral nanostructured surfaces.

## 3 Fabrication methods for nanostructured antiviral surfaces

The performance of nanostructured antiviral surfaces is largely determined by the controllability and uniformity of their structural morphology, which are directly governed by the fabrication methods. Currently, the primary approaches for constructing antiviral nanostructures include wet etching (Hasan et al., 2020a; Hasan et al., 2020b), template-assisted fabrication (Mah et al., 2026), reactive ion etching (Mah et al., 2024), and radio frequency sputtering (Shigetoh et al., 2025; Shigetoh et al., 2023). These methods differ in terms of structural precision, scalability, and material compatibility, thereby exerting a significant influence on the resulting antiviral performance.

### 3.1 Wet etching

**FIGURE 2:**
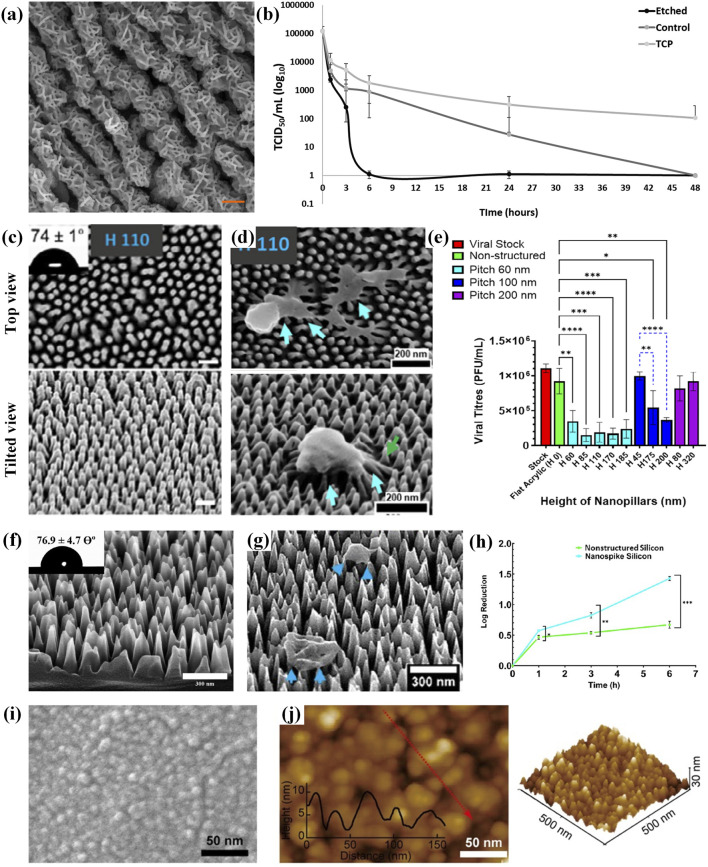
Fabrication and antiviral performance of nanostructured surfaces. **(a,b)** Wet-etched nanostructures and their antiviral activity against SARS-CoV-2. Reproduced with permission from ref (Hasan et al., 2020a). Copyright 2020 American Chemistry Society. **(c–e)** Template-assisted nanopillar arrays and corresponding hPIV-3 interaction and inactivation. Reproduced with permission from ref (Mah et al., 2026). Copyright 2026 Wiley-VCH GmbH. **(f–h)** Reactive ion etched nanospikes and time-dependent antiviral performance. Reproduced with permission from ref (Mah et al., 2024). Copyright 2023 American Chemistry Society. **(i,j)** RF-sputtered nanocolumnar structures. Reproduced with permission from ref (Shigetoh et al., 2023). Copyright 2023 American Chemistry Society.

Wet etching involves the selective chemical corrosion of material surfaces to generate nanoscale features or surface roughness, and is widely used for fabricating nanostructured surfaces. By tuning parameters such as solution composition, reaction time, and temperature, random or semi-ordered nanostructures can be produced. Common approaches include acid etching, alkaline etching, and oxidation-assisted etching. Hasan et al. (2020a) fabricated nanostructures with a characteristic size of ∼23 nm on Al 6,063 alloy via wet etching, achieving approximately a 5-log reduction in SARS-CoV-2 viral load within 6 h (Figures 2a,b).

### 3.2 Template-assisted fabrication

Template-assisted methods replicate predefined structures onto target surfaces using templates such as anodic aluminum oxide (AAO), nanoimprint molds, or biological templates, enabling the fabrication of large-area and ordered nanostructures. Common techniques include nanoimprint lithography and soft lithography. Mah et al. (2026) used AAO templates in ultraviolet nanoimprinting to fabricate nanopillar arrays and systematically investigated their antiviral performance, demonstrating that a dense array with a 60 nm pitch achieved approximately a 1.2-log reduction in hPIV-3 infectivity within 1 h (Figures 2c–e).

### 3.3 Reactive ion etching

Reactive ion etching (RIE) is a plasma-based dry etching technique that enables anisotropic surface structuring by introducing reactive gases under low pressure and applying an electric field. By adjusting parameters such as gas composition (e.g., CF_4_, SF_6_, Cl_2_), power, and etching duration, highly controllable nanostructures (such as nanopillars, nanoneedles, and high-aspect-ratio features) can be fabricated. Mah et al. (2024) employed RIE to produce sharp nanoneedle structures on silicon (tip diameter ∼2 nm, height ∼290 nm), which exhibited enhanced antiviral activity against human parainfluenza virus type 3 (hPIV-3), achieving approximately a 1.5-log reduction in infectivity within 6 h (Figures 2f–h).

### 3.4 Radio frequency sputtering

Radio frequency (RF) sputtering is a plasma-assisted thin-film deposition technique in which ions generated in a low-pressure plasma bombard a target material, ejecting atoms that subsequently deposit onto a substrate. By controlling parameters such as RF power, gas composition (e.g., Ar or reactive gases), and deposition time, the composition, thickness, and microstructure of the resulting films can be finely tuned, enabling the formation of nanostructured coatings. Shigetoh et al. (2023) fabricated nanocolumnar Cu and Cu oxide films via sputtering, producing biomimetic nanostructured surfaces (Figures 2i,j). The nanocolumnar films exhibited the highest antiviral activity, achieving approximately a 5-log reduction in viral load within 30 min, along with good oxidation resistance.

In addition, laser-based surface structuring has been widely used for surface functionalization because of its mask-free processing capability, high flexibility, and precise controllability over micro/nanostructures (Stratakis et al., 2020; Sun et al., 2026; Yang et al., 2026). By tuning parameters such as laser wavelength, pulse duration, fluence, and scanning strategy, various functional surface morphologies including laser-induced periodic surface structures (LIPSS), microcones, and hierarchical micro/nanostructures can be generated on metals, semiconductors, and polymers. Although laser-structured surfaces have not yet been extensively applied in antiviral studies, their ability to precisely tailor surface topography suggests considerable potential for future antiviral surface engineering and multifunctional biomedical applications (Nagarajan et al., 2021).

In summary, different preparation methods have distinct characteristics in terms of structural controllability, scalability, and cost. Wet etching is suitable for low-cost large-scale production but has limited controllability. RIE can achieve high-precision structures but has a higher cost. Template replication offers good consistency but is limited in flexibility. Radio frequency sputtering is suitable for functional coatings but complex structures require post-treatment. Laser-based processing provides high flexibility and precise regulation of hierarchical structures, demonstrating promising potential for future antiviral surface fabrication. Therefore, depending on specific application requirements, selecting or combining multiple fabrication strategies may further optimize surface structures and antiviral performance.

## 4 Surface composition and its contribution to antiviral performance

Although virus membrane deformation and penetration induced by nanostructures are considered the core mechanisms underlying physical antiviral effects, an increasing number of studies have demonstrated that surface composition also plays a significant role in determining antiviral performance (Birkett et al., 2022; Bregnocchi et al., 2022). For some antiviral surfaces, the overall antiviral efficacy arises from the synergy between surface morphology and material composition, rather than being solely governed by either nanostructures or chemical components alone (Shigetoh et al., 2025; Zhou et al., 2021).

Different material components can enhance the antiviral activity of nanostructured surfaces through multiple pathways. Metal and metal oxide materials (such as Ag, Cu, ZnO, and TiO_2_) can release metal ions or generate reactive oxygen species, thereby disrupting the viral envelope, denaturing viral proteins, and damaging genetic material, ultimately leading to virus inactivation (Rakowska et al., 2021; Tian et al., 2022). Carbon-based materials (such as graphene, graphene oxide, and carbon nanotubes) possess a high specific surface area and strong adsorption capability, which facilitates the enrichment of viral particles on the surface and may further damage the viral envelope through sharp edges or structural defects (Ye et al., 2015; Innocenzi and Stagi, 2020). In addition, functionalized polymers can modulate surface charge, wettability, and antifouling properties, thereby influencing virus–surface interactions and enhancing antiviral performance (Mouritz et al., 2021; Akbari et al., 2022).

Importantly, material composition often acts synergistically with nanostructures. Nanostructure-induced membrane deformation and penetration can compromise the integrity of the viral envelope, rendering viruses more susceptible to metal ions, reactive oxygen species, and other active species. Meanwhile, functional materials can compensate for the limited virus capture and sustained inactivation associated with purely mechanical effects. Therefore, both surface morphology and material composition should be considered in the design of antiviral nanostructured surfaces. The rational integration of structural features and functional materials can enhance antiviral efficiency and provide continuous, broad-spectrum, and multi-mechanistic antiviral activity. Future antiviral surfaces are expected to evolve from single-component designs toward optimized structure–composition synergy for improved performance and stability.

## 5 Conclusions and future outlook

In conclusion, nanostructure-mediated inactivation of enveloped viruses provides a promising physical route for the development of antiviral surfaces based on interfacial interactions. This process is fundamentally governed by membrane stretching and localized stress concentration induced by multivalent contact, leading to disruption of the viral envelope, with structural spacing and array-induced synergy playing dominant roles.

The geometric parameters of nanostructures, including spacing, tip geometry, and height, significantly influence antiviral performance, while virus size and environmental conditions further modulate these effects. Although various fabrication approaches, such as etching, template-assisted methods, and plasma-based techniques, have enabled the construction of diverse nanostructures, challenges remain in achieving large-area, cost-effective, and highly uniform fabrication. Moreover, increasing evidence suggests that antiviral efficacy is often governed by the synergistic effects of surface topography and material composition, rather than by either factor alone.

Despite recent progress, significant challenges remain, including the incomplete understanding of nanostructure–virus interactions, the lack of well-established structure–composition–activity relationships, and the absence of standardized evaluation protocols. In addition, issues related to large-scale manufacturing, long-term stability, and biosafety must be addressed before practical implementation can be achieved. Future research should focus on integrating experimental, theoretical, and computational approaches to elucidate the mechanisms of viral inactivation, establish predictive design principles, and optimize the synergy between nanostructured features and functional materials. Such efforts will facilitate the rational design and scalable fabrication of next-generation antiviral surfaces with enhanced efficiency, durability, and practical applicability.
